# Precipitation Regulates Phenological Responses of Annual and Perennial Forbs to Warming and Degradation in an Alpine Meadow on the Qinghai–Xizang Plateau

**DOI:** 10.1002/ece3.74423

**Published:** 2026-09-29

**Authors:** Lanying Chen, Shujuan Cui, Jianping Sun, Wangwang Lv, Jingya Lv, Fang Yuan, Yang Zhou, A. Wang, Shiping Wang

**Affiliations:** ^1^ School of Ecology and Environment Xizang University Lhasa Xizang China; ^2^ College of Life Sciences China West Normal University Nanchong Sichuan China; ^3^ School of Resources and Environment Anqing Normal University Anqing Anhui China; ^4^ State Key Laboratory of Tibetan Plateau Earth System, Environment and Resources, Institute of Tibetan Plateau Research Chinese Academy of Sciences Beijing China; ^5^ University of the Chinese Academy of Sciences Beijing China

**Keywords:** Alpine meadow, experimental warming, grassland degradation, Phenological stages, soil temperature‐moisture decoupling, Tibetan plateau

## Abstract

Alpine grasslands on the Qinghai‐Xizang Plateau are increasingly threatened by climate warming and degradation, which promote an increase in annual plants in the community. However, how they shape the plant phenophases of annual and perennial plants remains unclear. In particular, there is limited understanding of whether and how ambient climate regulates their responses to warming and degradation. We conducted a three‐year warming experiment across moderately (MD) and severely degraded (SD) grasslands to determine the responses of phenophases of an annual and a perennial forb. We found that neither warming nor degradation significantly affected the onsets and durations of phenological sequences for the annual plant due to the offsetting of a direct advancing effect by an indirect delaying effect via decreased plant richness, which varied with the year. Warming effects on the onset of leaf‐out and end of leaf‐coloring of the perennial plant varied with degradation and year, whereas SD advanced the onset of leaf‐out and prolonged its total activity period relative to MD under warming. This suggested that warming could benefit the growth of perennial plants in the degraded alpine grasslands. Precipitation regulated the responses of phenophases of both annual and perennial plants to warming and degradation via decreased plant richness and/or increased their relative coverage in the community. Our findings indicate that attention should not only be paid to the life history characteristics of plants but also to the regulatory roles of vegetation status and ambient climate when evaluating the impact of climate change on plant phenology.

## Graphical abstract

Warming and degradation had negligible multi‐year mean effects on annual plant phenology, but their impacts varied strongly with interannual precipitation and spring soil moisture. Degradation amplified warming‐induced advancement of leaf‐out and extended the total active period of the perennial *Aster tataricus* via microclimatic amplification and positive dominance feedbacks. Precipitation played an overriding role, directly advancing the annual's phenology but delaying the perennial's leaf‐out. Life‐history strategies determine phenological sensitivity to warming under degraded conditions, with precipitation as a critical regulator. These findings have implications for predicting community dynamics and guiding restoration of degraded alpine grasslands under future climate change.

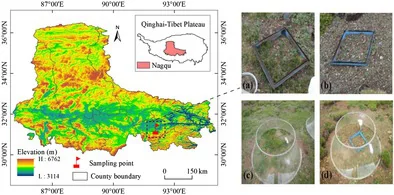

## 1 Introduction

Grassland ecosystems account for approximately 30% of the Earth's ice‐free terrestrial surface (Zhang et al. 2025; Gao et al. 2025). Climate change and degradation have profoundly impacted their structure and functions (Wang et al. 2022), which is potentially related to changes in plant phenology (Wolf et al. 2017; Shen et al. 2022). Plant phenology is one of the most sensitive indicators of responses to environmental changes (Wolkovich et al. 2012; Li et al. 2016). To date, studies have paid more attention to the impact of warming on the phenology of grassland plants (Wolkovich et al. 2012; Robinson et al. 2018; Sun et al. 2026), whereas studies on the impact of degradation remain scarce (Cui et al. 2017; Zhang et al. 2020). Degradation, in particular, reduces the proportion of perennial plants while increasing that of annual plants (Baer and Blair 2008; Cui et al. 2017; Wang et al. 2022). However, there is a lack of empirical research on which annual and perennial plants are more sensitive to warming in terms of phenology under degradation. This gap strongly restricts our ability to evaluate whether warming is conducive to the recovery of degraded grasslands from the perspective of phenological changes (Sun et al. 2025).

Studies show that warming advances the greening‐up and flowering of perennial plants, delays their coloring, and extends the durations of reproduction and the growing season (Wolkovich et al. 2012; Wang et al. 2014; Li et al. 2016; Shen et al. 2022; Chen et al. 2025). However, drought or decrease in soil moisture induced by warming (Dorji et al. 2013; Hu et al. 2020; Liu et al. 2021; Sun et al. 2026), and increased nitrogen (N) availability induced by warming (Jiang et al. 2018; Zhang et al. 2020) or N addition (Wang and Tang 2019; Yang et al. 2023; Zhang et al. 2026) may all mitigate the aforementioned warming effect. Moreover, degraded grasslands have reduced soil nutrient availability and plant diversity (Wang et al. 2012; Shi et al. 2026) which constrain plant growth (Shi et al. 2026), thereby affecting the phenology of perennial plants (Wolf et al. 2017; Zhang et al. 2020). In addition to temperature, water availability—primarily precipitation—critically regulates plant phenology on the Qinghai‐Xizang Plateau, where water limitation pervasively constrains plant growth. Precipitation can directly modulate soil moisture and nutrient availability, and interact with warming to either amplify or buffer its phenological effects: increased water may alleviate stress and permit warming‐induced advances, while drought can override thermal effects and delay phenological events (Ganjurjav et al. 2020). Indeed, a manipulative experiment at Nagqu showed that warming delayed green‐up in warm‐dry years but advanced it in cold‐wet years, with early‐season precipitation offsetting warming‐induced delays in dry years (Ganjurjav et al. 2020). At the regional scale, preseason precipitation exerts stronger control over green‐up in drier areas, whereas warming dominates in relatively moist regions (Shen et al. 2022). Moreover, a global meta‐analysis confirmed that functional group and aridity jointly determine phenological responses to climate change (Sun et al. 2026). These findings underscore that water availability critically mediates temperature‐driven phenological shifts—an insight vital given concurrent projected changes in both temperature and precipitation on the Plateau.

Yet, most of this evidence has focused on perennial‐dominated communities, leaving a critical gap in our understanding of how annual plants—which often proliferate in degraded grasslands—respond to the interactive effects of warming and water availability (Cui et al. 2017). Indeed, warming has been shown to exert only weak direct effects on the phenology of annuals in degraded systems, while primarily enhancing their reproductive output through increased plant height (Cui et al. 2017). This muted phenological response of annuals is largely attributable to the fact that their germination timing is strongly driven by interannual variation in precipitation or pre‐germination soil moisture, rather than by temperature itself (Facelli and Chesson 2008; Mayfield et al. 2014; Cui et al. 2017). Thus, the impact of warming on plant phenology is not unidirectional but depends on a suite of interacting factors, including precipitation or soil moisture conditions (Sun et al. 2026), soil nutrient availability (Wang and Tang 2019; Zhang et al. 2026), and plant diversity (Wolf et al. 2017). Unlike perennial plants, which can maintain population size through vegetative propagation, annual plants may complete their entire life history in the growing season and maximize seed production to ensure population persistence (Sun et al. 2025). Thus, the different life history characteristics of annual and perennial plants may determine their distinct phenological responses to warming and grassland degradation (Cleland et al. 2006; Cui et al. 2017; Zhang et al. 2020). However, to date, few empirical experiments have simultaneously explored the phenological responses of annual and perennial plants to warming. In particular, it is still unclear whether and how grassland degradation regulates annual and perennial plants' phenological responses to warming.

Here, we conducted a 3‐year study using common annual ( *Chenopodium glaucum* ) and perennial ( *Aster tataricus* ) plants chosen from moderately degraded and severely degraded grasslands on the Qinghai‐Tibetan Plateau. The two factorial factors experiment included manipulative warming using open‐top chambers (OTC) (no warming vs. warming) with degradation status (moderate degradation vs. severe degradation). Our objectives were to determine: (1) How do warming and degradation affect the phenophases of annual and perennial alpine plants? (2) How does ambient climate regulate their responses to warming and degradation? and (3) What are the main factors affecting shifts of their phenophases and the underlying mechanisms? We hypothesized that (1) the phenological responses of perennial plants could be more sensitive to warming relative to annual plants; (2) degradation could amplify their responses to warming; and (3) the responses of phenology could be modulated by ambient climate in the alpine grasslands.

## 2 Materials and Methods

### 2.1 Study Location

The experiment was conducted from 2014 to 2016 at the Naqu Alpine Meadow Ecosystem National Field Observation and Research Station (field station) in Tibet, China, on the central Tibetan Plateau (31°16′ N, 92°06′ E; 4453 m a.s.l.) (Figure 1). This region exhibits a harsh alpine climate with a mean annual temperature of −2.1°C and annual precipitation of 430 mm (80% occurring in June–September). *Kobresia pygmaea* is a dominant species, forming 80%–90% cover in the non‐degraded grassland community (Miehe et al. 2008). Moderately degraded grassland (MD) had approximately 30% native vegetation cover, and severely degraded grassland (SD) was desertified without native vegetation (Figure 1). Annual *C. glaucum* and perennial *A. tataricus* were common dominant plant species in the MD and SD grasslands, and were absent in non‐degraded grassland.

**FIGURE 1:**
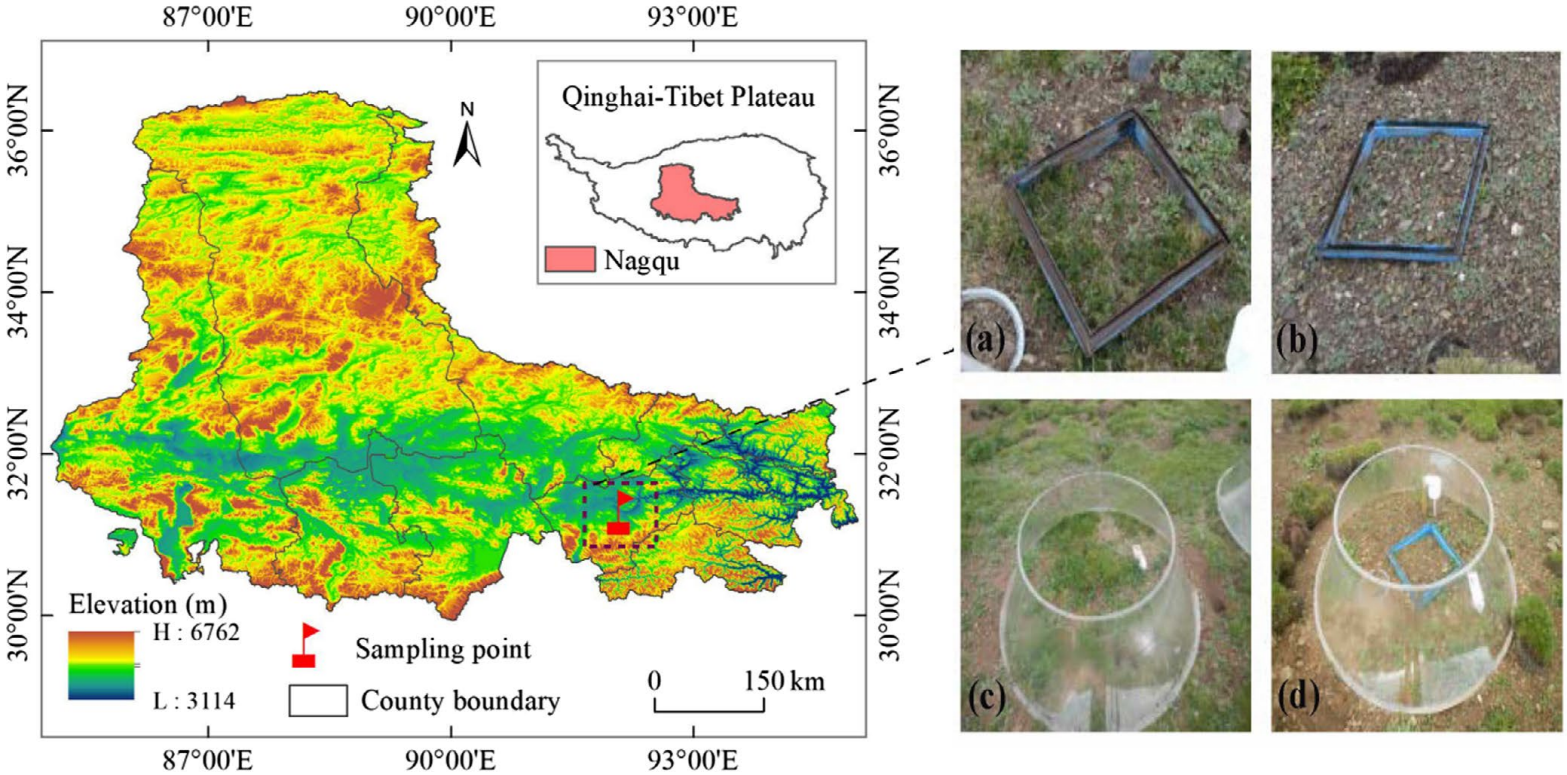
Experimental site of the OTC warming treatments at different degradation levels. OTC, Open‐top chamber. (a) no warming with moderate degradation (NWMD); (b) no warming with severe degradation (NWSD); (c) warming with moderate degradation (WMD); (d) warming with severe degradation (WSD).

### 2.2 Experimental Design

A two‐factorial experiment was conducted using passive warming of open‐top chambers (OTCs: 1.5 m base diameter, 1.0 m top diameter, 0.5 m height) with two degradation levels (MD and SD). In total, there were four treatments: no warming with moderate degradation (NWMD), no warming with severe degradation (NWSD), warming with moderately degradation (WMD), and warming with severe degradation (WSD) (Figure 1). Each treatment had four replicates. Two plants of annual *C. glaucum* and perennial *A. tataricus* were chosen from each plot. *C. glaucum* and *A. tataricus* were selected as focal species because they are common dominant forbs in moderately and severely degraded grasslands on the central Qinghai‐Xizang Plateau, and both are absent from non‐degraded communities (Cui et al. 2017; Zhang et al. 2020). These two species represent contrasting life‐history strategies— *C. glaucum* is an annual that completes its entire life cycle within a single growing season and reproduces exclusively via seeds, whereas *A. tataricus* is a perennial that can propagate vegetatively and reproduce over multiple years—making them ideal model species for comparing phenological responses between functional groups.

### 2.3 Measurements of Environmental Factors

Air temperature and precipitation data were provided by the field station. Soil temperature and soil moisture at 10 cm depth were measured automatically using EM50 data loggers (Decagon Devices Inc., USA). Data were recorded every hour as the average of 60 measurements per hour. Due to limited EM50 equipment, soil sensors were installed for four plots within the OTC chamber on the MD and SD grasslands, while only two plots were installed with soil sensors for the no warming treatments outside the OTC on the MD and SD grasslands. For air temperature and humidity, sensors were installed in all four plots within the OTC chamber, whereas only one sensor was installed to measure the air temperature and humidity outside the OTC on the MD and SD grasslands. During the study period (2014–2016), annual precipitation ranged from 280.4 to 406.7 mm, and mean annual air temperature varied between 0.0°C and 0.7°C, with 2015 identified as a drought year (Figure S1). Degradation and warming exerted synergistic effects on soil microclimate: warming increased soil temperature more strongly in SD plots (+1.08°C) than MD plots (+0.69°C), while its moisture‐suppressing effect was attenuated under SD (15.4%–18.8% reduction vs. 45.9%–49.1% in MD plots) (Zhou et al. 2024).

### 2.4 Soil Physicochemical Analysis

On August 12, 2016, soil samples were collected from the 0–10 cm soil in each plot using a 5 cm diameter auger. The samples were homogenized, passed through a 2 mm sieve, and roots and other plant debris were carefully removed prior to analysis. Soil texture was determined using the hydrometer method (Ashworth et al. 2001). Total organic carbon (TOC) was measured using a TOC‐5000A analyzer (Shimadzu Corp., Kyoto, Japan). Soil total nitrogen (TN), total phosphorus (TP), and total potassium (TK) were analyzed by isotope ratio mass spectrometry (Isoprime‐EuroEA 3000, Milan, Italy). Soil pH was determined in a 1:2 (w/v) soil–to–deionized water suspension. The concentrations of available nitrogen (AN), available phosphorus (AP), and available potassium (AK) were quantified following the procedures described by Wu et al. (2011). Principal component analysis (PCA) was applied to soil physicochemical properties due to strong multicollinearity among them (|*r*| > 0.7, Figure S2a). The first principal component (SoilPC1) explained 92% of the variance and represented an integrated fertility gradient (Figure S2b).

### 2.5 Measurements of Plant Phenophases

The measurements of plant phenophases have been described in previous studies (Cui et al. 2017; Zhang et al. 2020). Briefly, to ensure consistent tracking, 10 individuals per species were marked within each plot by the end of 2013. Observations were made at an interval of 3 or 4 days from early April to the end of October in each year. For the annual plant, there were five phenological events, including start dates of germination (onset of leaf out, OLO), first budding (FB), first flowering (FF), first fruiting (FFR), and end of leaf coloring (ELC). Corresponding to these phenological events, the durations of each phenophase (i.e., leaf duration (DL), budding duration (DB), flowering duration (DF), fruiting duration (DFR), and leaf coloring duration (DLC)) were defined as the differences between their end dates and first dates (Li et al. 2016). For the perennial plant, their reproductive phases were absent, and only their OLO and ELC were measured. The onset of a given phenological event were defined as the date when ≥ 15% of observed individuals within a plot had occurred, and its end was defined as the date when ≥ 95% of individuals had reached it. The total activity period (TAP) was calculated as the difference between ELC and OLO for each plot.

### 2.6 Measurements of Plant Community Composition and Diversity

In late August of each year, plant community composition was surveyed using a 50 × 50 cm quadrat subdivided into 100 grids (5 × 5 cm). At each grid intersection, species identity and plant height were recorded. Total vegetation cover was calculated as the proportion of grid intersections occupied by plants. Mean plant height was derived from all recorded individuals. Relative cover of each species was estimated as its individual count divided by the total number of individuals. Species richness was quantified as the total number of species per quadrat.

### 2.7 Statistical Analysis

All analyses were conducted in R 4.4.3 (RStudio). Soil physicochemical properties were analyzed by two‐way ANOVA with degradation and warming as fixed factors. Plant community composition was assessed via NMDS based on Bray–Curtis dissimilarity, with treatment differences tested by PERMANOVA (999 permutations). To account for repeated measurements across years, linear mixed‐effects models (LMMs) were employed for all annually measured response variables, including species richness, relative cover, and phenological events. In these models, plot was included as a random intercept, year was treated as a random factor, and degradation (D), warming (W), and their interaction (W × D) were fitted as fixed effects. Post hoc pairwise comparisons were conducted using Tukey's HSD test. Prior to structural equation modeling, soil physicochemical properties were reduced via PCA, extracting the first principal component (SoilPC1, explaining 92% of the variance; Figure S2). Pearson correlations informed path specifications. SEMs disentangled direct and indirect pathways linking warming, degradation, precipitation, soil properties (ST, SM, SoilPC1), community characteristics, and species‐specific phenological events. All models were evaluated using the chi‐square test (*χ* ^2^, *p* > 0.05, indicating good fit), Fisher's C statistic, and Akaike information criterion (AIC). Graphics were generated with ggplot2, ggtext, and semPlot.

## 3 Results

### 3.1 Responses of Phenological Events

#### 3.1.1 Three‐Year Mean Responses

Over the three‐year experimental period, warming, degradation, and their interaction had no significant effects on the mean onsets of most phenophases, end dates of leaf coloring, or durations of all phenophases for the annual *C. glaucum* (Figure 2a,c; Tables 1 and 2). By contrast, degradation and its interaction with warming significantly affected the phenology of the perennial *A. tataricus* (Figure 2b,d; Table 2). Specifically, severe degradation (SD) significantly advanced the mean onset of leaf‐out in *A. tataricus* under warming (*p* < 0.05; Figure 2b; Table 1).

**FIGURE 2:**
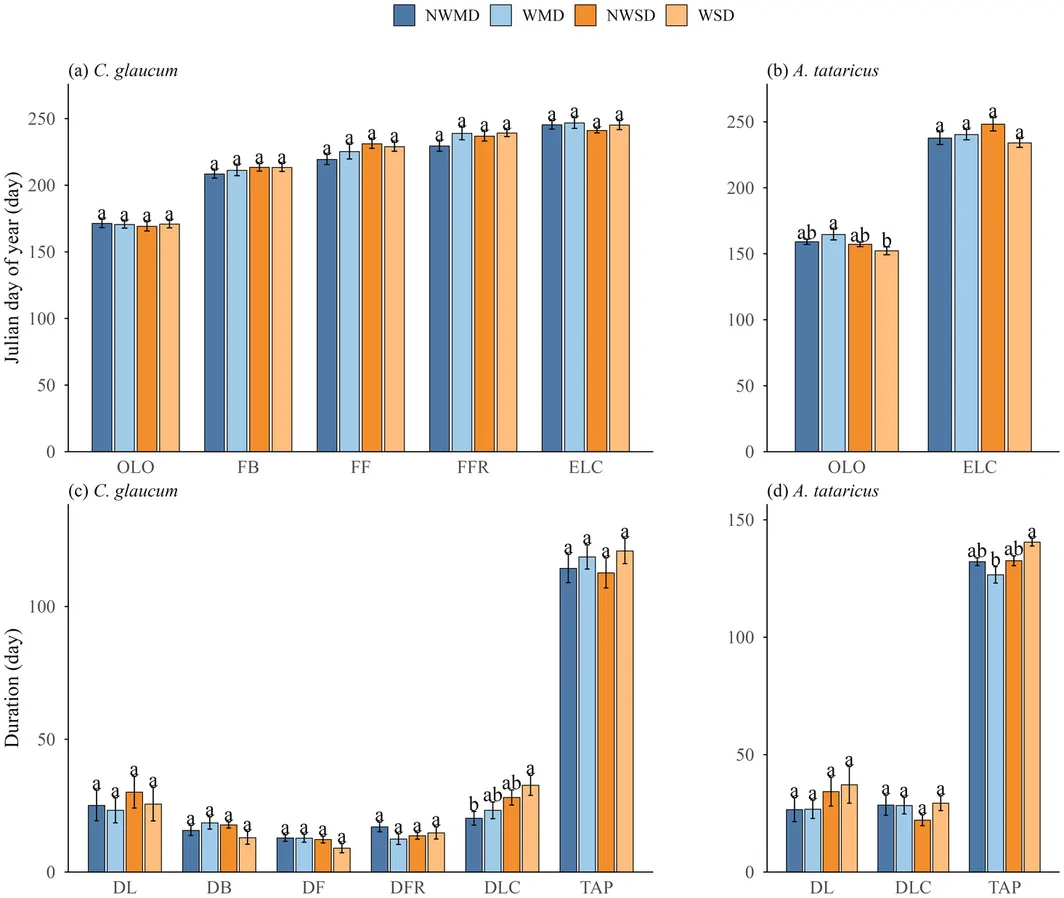
Phenological onset and duration responses to warming and degradation for *C. glaucum* (a, c) and *A. tataricus* (b, d) based on three–year means (2014–2016). Start dates of leaf‐out (OLO), first budding (FB), first flowering (FF), first fruiting (FFR), and end of leaf coloring (ELC) are shown for onset events; durations of leafing (DL), budding (DB), flowering (DF), fruiting (DFR), leaf coloring (DLC), and total active period (TAP) are shown for duration events. Different letters above bars indicate significant differences among treatments at *p* < 0.05.

**TABLE 1:** Results of linear mixed‐effects models (LMMs) testing the effects of degradation (D), warming (W), and their interaction (W × D) on phenological onset events of *C. glaucum* and *A. tataricus* .

Phenological event	Effect type	*C. glaucum*		*A. tataricus*	
		*F*	*p*	*F*	*p*
OLO	Year (Y)	63.282	**< 0.001**	91.011	**< 0.001**
OLO	Warming (W)	0.037	0.851	0.061	0.807
OLO	Degradation (D)	0.179	0.679	36.174	**< 0.001**
OLO	Y × W	1.923	0.168	14.038	**< 0.001**
OLO	Y × D	3.570	**0.044**	3.351	**0.046**
OLO	W × D	0.333	0.574	19.330	**< 0.001**
OLO	Y × W × D	2.511	0.102	1.798	0.180
FB	Y	70.311	**< 0.001**	—	—
FB	W	0.401	0.539	—	—
FB	D	2.894	0.115	—	—
FB	Y × W	0.752	0.482	—	—
FB	Y × D	1.351	0.278	—	—
FB	W × D	0.452	0.514	—	—
FB	Y × W × D	3.469	**0.047**	—	—
FF	Y	95.104	**< 0.001**	—	—
FF	W	0.382	0.548	—	—
FF	D	6.452	**0.026**	—	—
FF	Y × W	0.199	0.821	—	—
FF	Y × D	10.302	**< 0.001**	—	—
FF	W × D	1.774	0.208	—	—
FF	Y × W × D	13.492	**< 0.001**	—	—
FFR	Y	56.299	**< 0.001**	—	—
FFR	W	4.606	0.053	—	—
FFR	D	1.933	0.190	—	—
FFR	Y × W	1.318	0.286	—	—
FFR	Y × D	5.033	**0.015**	—	—
FFR	W × D	1.689	0.218	—	—
FFR	Y × W × D	2.051	0.150	—	—
ELC	Y	28.875	**< 0.001**	14.179	**< 0.001**
ELC	W	1.711	0.199	2.861	0.117
ELC	D	1.925	0.174	0.376	0.551
ELC	Y × W	1.287	0.289	2.943	0.072
ELC	Y × D	1.472	0.243	2.523	0.101
ELC	W × D	0.402	0.530	6.129	**0.029**
ELC	Y × W × D	2.000	0.150	1.975	0.161

*Note:* Significant *p*‐values (< 0.05) are shown in bold. Plot was included as a random intercept; year was treated as a random factor. “—” indicates not applicable ( *A. tataricus* lacked reproductive phenophases in this study).

Abbreviations: D, degradation (moderately degraded, MD; severely degraded, SD); ELC, end of leaf coloring; FB, first budding; FF, first flowering; FFR, first fruiting; OLO, onset of leaf‐out; W, warming (no‐warming, NW; warming, W).

**TABLE 2:** Results of linear mixed‐effects models (LMMs) testing the effects of degradation (D), warming (W), and their interaction (W × D) on phenological duration events of *C. glaucum* and *A. tataricus* .

Phenological event	Effect type	*C. glaucum*		*A. tataricus*	
		*F*	*p*	*F*	*p*
DL	Y	240.059	**< 0.001**	283.153	**< 0.001**
DL	W	3.660	0.064	0.777	0.395
DL	D	4.907	**0.033**	26.730	**< 0.001**
DL	Y × W	0.993	0.380	0.581	0.567
DL	Y × D	2.149	0.131	18.867	**< 0.001**
DL	W × D	0.649	0.426	0.618	0.447
DL	Y × W × D	2.358	0.109	4.962	**0.016**
DB	Y	1.054	0.359	—	—
DB	W	0.398	0.532	—	—
DB	D	1.219	0.277	—	—
DB	Y × W	3.602	**0.037**	—	—
DB	Y × D	9.002	**< 0.001**	—	—
DB	W × D	5.851	**0.021**	—	—
DB	Y × W × D	4.322	**0.021**	—	—
DF	Y	2.606	**0.095**	—	—
DF	W	1.083	0.319	—	—
DF	D	1.829	0.201	—	—
DF	Y × W	0.044	0.957	—	—
DF	Y × D	3.740	**0.039**	—	—
DF	W × D	0.977	0.342	—	—
DF	Y × W × D	0.013	0.987	—	—
DFR	Y	4.923	**0.016**	—	—
DFR	W	0.747	0.404	—	—
DFR	D	0.061	0.809	—	—
DFR	Y × W	2.473	0.106	—	—
DFR	Y × D	1.418	0.262	—	—
DFR	W × D	1.958	0.187	—	—
DFR	Y × W × D	0.802	0.460	—	—
DLC	Y	8.844	**< 0.001**	15.703	**< 0.001**
DLC	W	2.954	**0.094**	2.686	0.110
DLC	D	15.287	**< 0.001**	1.571	0.218
DLC	Y × W	4.155	**0.024**	3.931	0.029
DLC	Y × D	6.782	**0.003**	16.994	**< 0.001**
DLC	W × D	0.129	0.722	2.945	0.095
DLC	Y × W × D	6.315	**0.004**	0.313	0.733
TAP	Y	108.464	**< 0.001**	8.016	**0.002**
TAP	W	5.238	**0.041**	0.278	0.607
TAP	D	0.011	0.917	10.508	**0.007**
TAP	Y × W	10.133	**< 0.001**	0.725	0.495
TAP	Y × D	4.536	**0.021**	0.634	0.539
TAP	W × D	0.507	0.490	9.322	**0.010**
TAP	Y × W × D	24.700	**< 0.001**	2.532	0.101

*Note:* Significant *p*‐values (< 0.05) are shown in bold. Plot was included as a random intercept; year was treated as a random factor. “—” indicates not applicable.

Abbreviations: D, degradation (moderately degraded, MD; severely degraded, SD); DB, budding duration; DF, flowering duration; DFR, fruiting duration; DL, leaf duration; DLC, leaf‐coloring duration; TAP, total active period; W, warming (no‐warming, NW; warming, W).

#### 3.1.2 Interannual Variation in Phenological Responses

Phenological responses of *C. glaucum* varied considerably across years (Figure 3a,c; Tables 1 and 2; Tables S3 and S4). In 2014, warming marginally delayed first fruiting by 14.8 days under moderate degradation (*p* = 0.053, Table 1; Table S3). In 2015, both WMD and WSD significantly delayed first fruiting by 5.3–11.3 days compared with NWMD (Figure 3a,c; Table S3). In 2016, WMD significantly advanced first budding by 7.8 days relative to NWSD and by 10.5 days relative to WSD, and advanced first flowering by 17.0 days relative to NWSD and by 24.8 days relative to WSD (Figure 3a, Table 1; Table S3).

**FIGURE 3:**
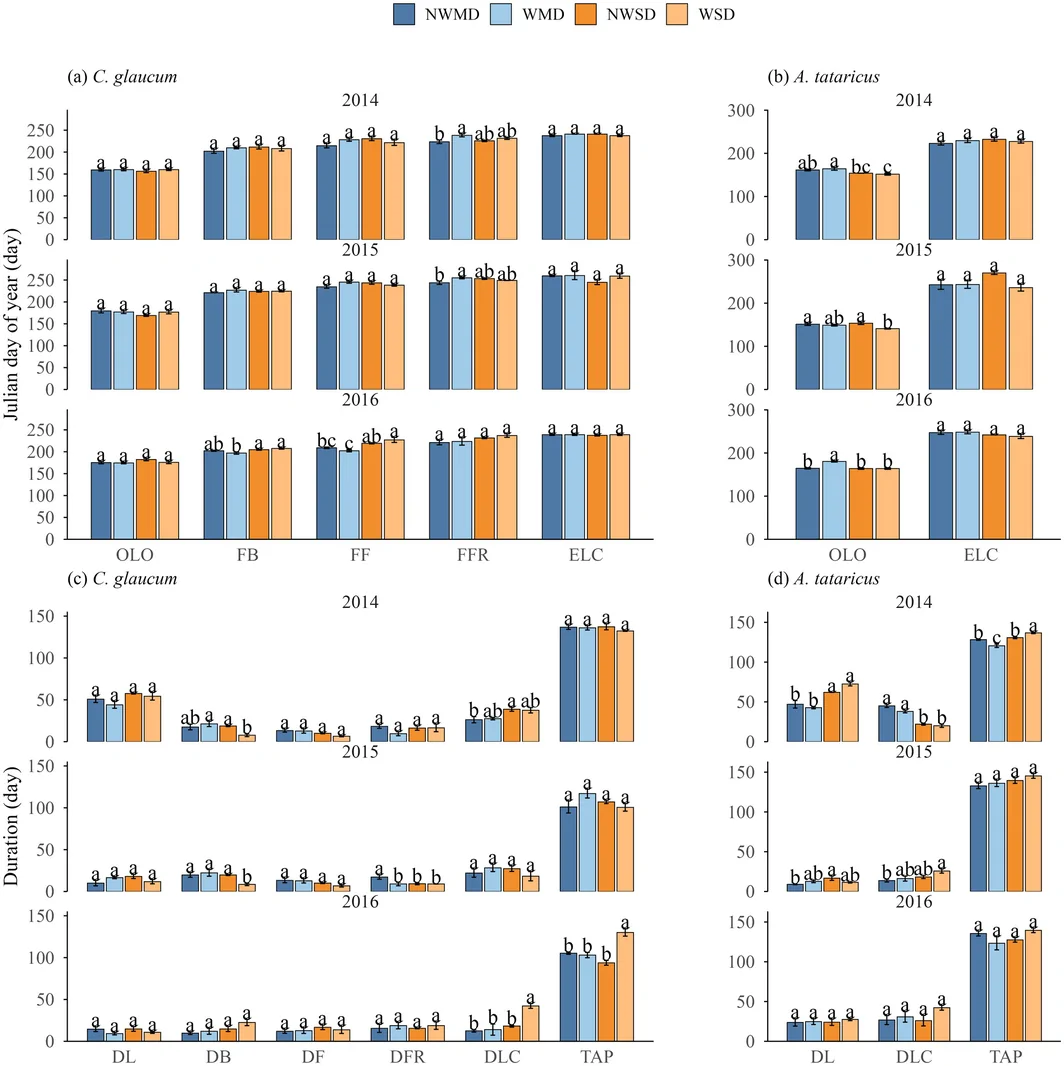
Phenological onset and duration responses to warming and degradation in *C. glaucum* (a, c) and *A. tataricus* (b, d) during 2014–2016. Start dates of leaf‐out (OLO), first budding (FB), first flowering (FF), first fruiting (FFR), and end of leaf coloring (ELC) are shown for onset events; durations of leafing (DL), budding (DB), flowering (DF), fruiting (DFR), leaf coloring (DLC), and total active period (TAP) are shown for duration events. Different letters above bars indicate significant differences among treatments at *p* < 0.05.

Treatment effects on phenological durations of *C. glaucum* also varied among phenophases and years (Table 2; Table S4). Budding duration was significantly affected by the warming × degradation interaction (*p* = 0.021), with WSD shortening it by 9.7 days in 2014 and by 11.3 days in 2015 compared with NWMD. In 2015, WSD and NWSD significantly shortened fruiting duration by 8.0–8.2 days relative to NWMD (Table S4). SD prolonged leaf‐coloring duration by 11.3–12.5 days relative to MD regardless of warming in 2014 (*p* < 0.001, Table 2; Table S4), and prolonged it by 28.2 days compared with MD under warming in 2016 (Table S4). In 2016, SD shortened the total active period by 11.4 days relative to MD under no warming, whereas warming prolonged it by 36.2 days compared with no warming under SD; SD prolonged TAP by 27.0 days compared with MD under warming (Figure 3c; Table S4).

For *A. tataricus* , warming alone did not affect the mean onset of leaf‐out or the end date of leaf coloring. However, degradation and its interaction with warming significantly advanced leaf‐out under warming, with strong interannual variation (Figure 2b; Table 1; Table S3). SD advanced leaf‐out by 7.5 days relative to MD under no warming, by 12.4 days under warming in 2014, and by 16.5 days under warming in 2016 (*p* < 0.001, Figure 3b; Table S3).

Treatment effects on phenological durations of *A. tataricus* also varied with phenophase and year (Table 2; Table S4). SD prolonged leaf duration by 15.0 days under no warming (NWSD vs. NWMD) and by 29.7 days under warming (WSD vs. WMD) in 2014 (Figure 3d; Table S4), but significantly shortened leaf‐coloring duration by 16–25 days relative to MD regardless of warming in 2014 (Figure 3d; Table S4). SD prolonged leaf duration by 7.8 days under no warming (NWSD vs. NWMD) in 2015. The total active period was significantly influenced by the warming × degradation interaction (*p* = 0.010, Table 2; Table S4). WSD prolonged the mean total active period by 13.9 days relative to WMD over the experimental period and by 16.3 days under warming (WSD vs. WMD) in 2014 (Figure 3d; Table S4).

### 3.2 Responses of Soil Physicochemical Properties

Warming had no significant effect on any of the measured soil physicochemical properties (all *p* > 0.05; Figure S3; Table S1). By contrast, degradation degree exerted a highly significant influence on all soil variables (all *p* < 0.001; Figure S3; Table S1). Specifically, severely degraded meadows (SD) exhibited significantly higher soil pH (+11.0%) and substantially lower levels of total organic carbon (−45.5%), total nitrogen (−76.9%), available nitrogen (−58.9%), total phosphorus (−66.6%), available phosphorus (−58.1%), total potassium (−44.5%), and available potassium (−33.4%) than moderately degraded meadows (MD). No significant warming × degradation interactions were detected for any soil properties (all *p* > 0.05; Table S1).

### 3.3 Responses of the Plant Community

Non‐metric multidimensional scaling (NMDS) ordination revealed clear separation in plant community structure among treatments (Stress = 0.15; Figure 4a), with PERMANOVA attributing 57.3% of the observed variation to the warming × degradation interaction (*F* = 15.21, *p* = 0.001; Table S2). Warming reduced species richness by 17.6% (*p* = 0.045; Table S2), while SD reduced it by 54.6% relative to MD (*p* < 0.001; Table S2), with a significant warming × degradation interaction (*p* = 0.019; Figure 4b; Table S2). SD significantly increased the relative cover of *A. tataricus* by 173.5% compared with MD (Figure 4c; Table S2), whereas warming increased the relative cover of *C. glaucum* by 66.5% (Figure 4d; Table S2), with degradation showing a marginal effect (*F* = 3.87, *p* = 0.053).

**FIGURE 4:**
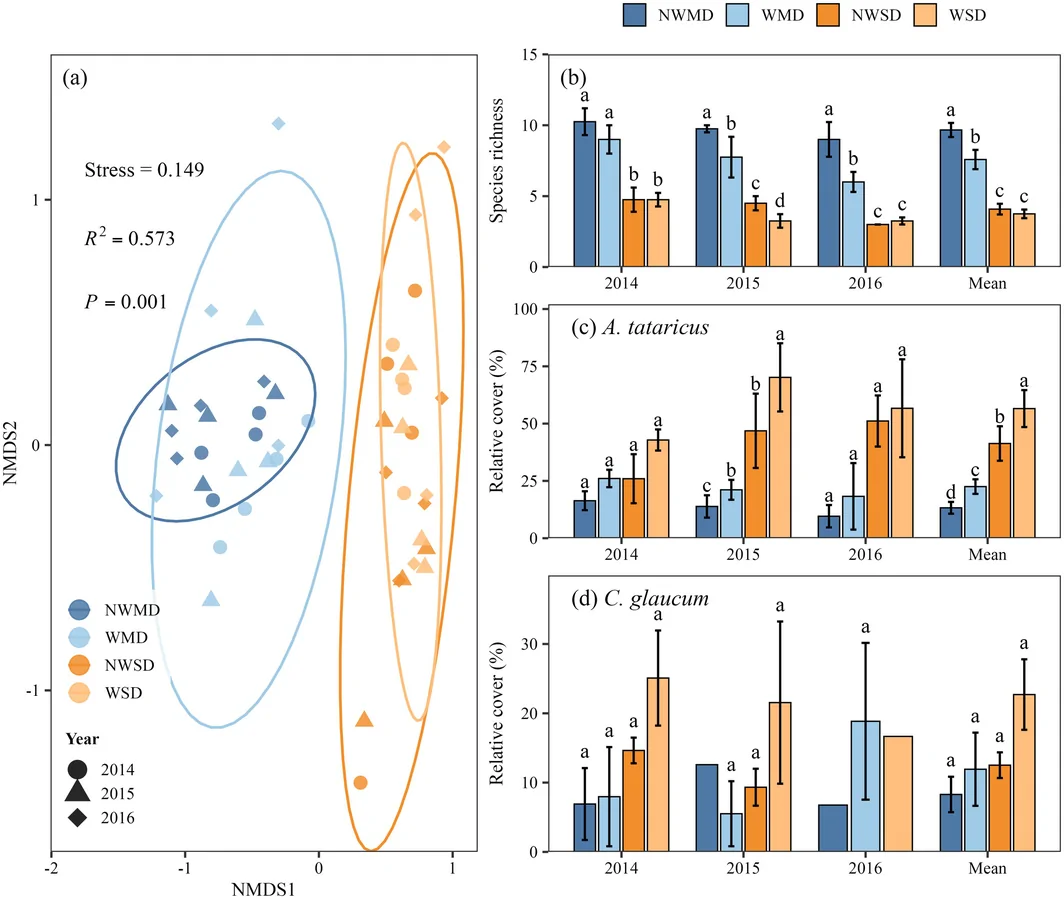
Effects of experimental warming and grassland degradation on (a) plant community composition (NMDS), (b) species richness, (c) relative cover of *A. tataricus* , and (d) relative cover of *C. glaucum* . Mean ± SE are presented in the figure; different lowercase letters indicate significant differences among treatments at the *p* < 0.05 level.

### 3.4 Factors Affecting Phenophases

For *C. glaucum* (Figure 5a), mean annual precipitation (MAP) was significantly negatively correlated with first budding, first flowering, first fruiting, and end of leaf coloring. Soil moisture was significantly negatively correlated with the onset of fruiting. Additionally, all onsets of phenophases were significantly positively correlated with each other. For duration metrics, SoilPC1 (integrated soil fertility) was significantly negatively correlated with leaf‐coloring duration, while MAT was significantly negatively correlated with leaf‐coloring duration. Total active period was significantly positively correlated with both leaf duration and leaf‐coloring duration.

**FIGURE 5:**
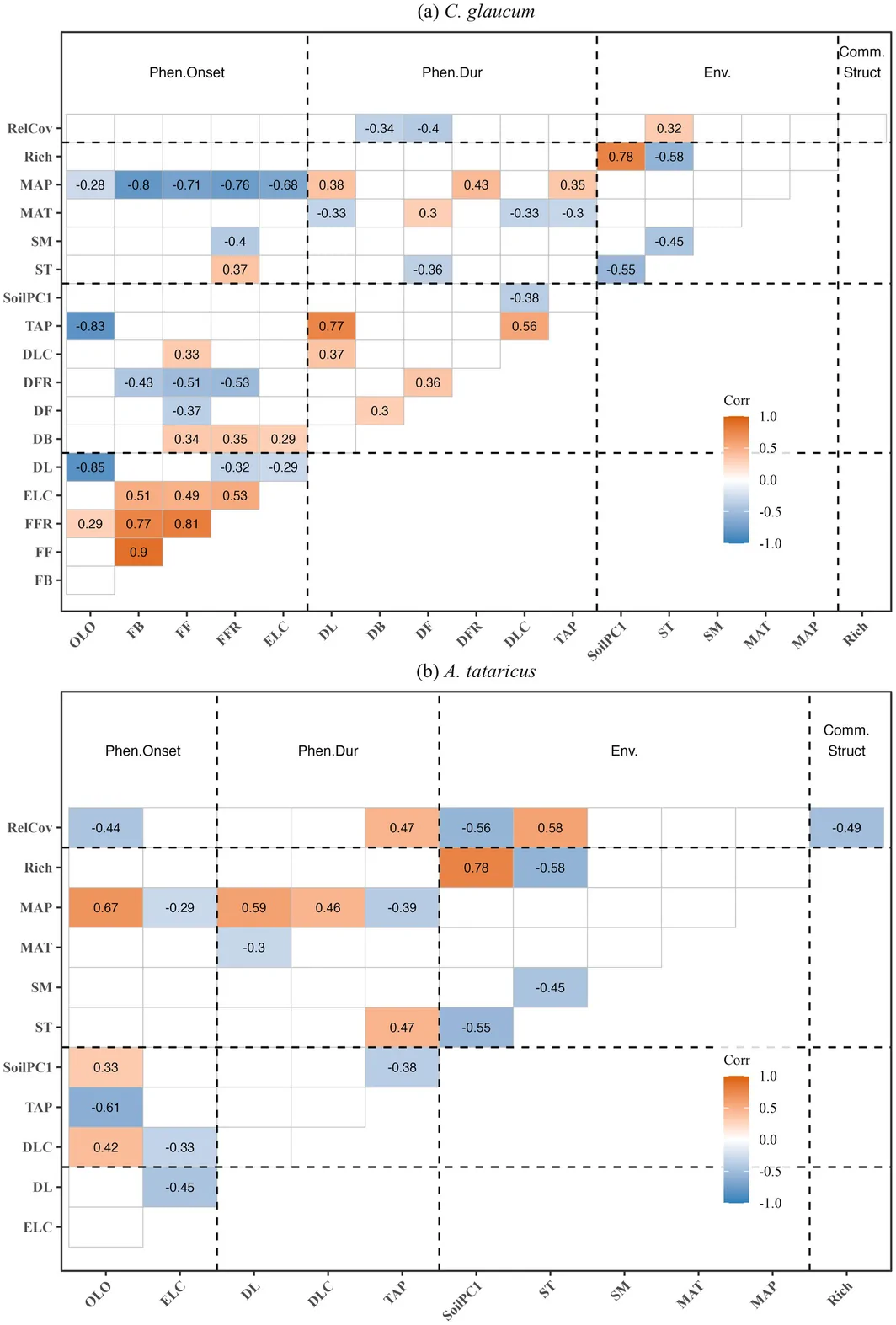
Correlation matrices of driving factors, environmental factors, community characteristics, and phenological traits in *C. glaucum* and *A. tataricus* . Colors represent Pearson's *r*; only significant correlations (*p* < 0.05) are shown. Deg, degradation; MAP, mean annual precipitation; MAT, mean annual temperature; Rich, species richness; RelCov, relative cover; SM, soil moisture; SoilPC1, soil principal component 1; ST, soil temperature; Warm, warming.

For *A. tataricus* (Figure 5b), MAP was significantly positively correlated with the onset of leaf‐out, leaf duration, and leaf coloring duration. The relative cover of *A. tataricus* was significantly negatively correlated with leaf‐out but positively correlated with total active period. Furthermore, the onset of leaf‐out was significantly positively correlated with leaf coloring duration.

### 3.5 Direct and Indirect Effects of Degradation and Warming on Phenological Events

For *C. glaucum* (Figure 6a,b), the total net effect of warming on the onset of leaf‐out (OLO) was small (0.02) due to the offsetting effects of a direct advance effect (−0.75) and an indirect delay effect via increased soil temperature (ST) (0.63) and a decrease in plant richness (0.14). The total net effect of degradation delayed OLO (0.25) through directly advanced OLO (−0.85) and indirectly delayed it via ST (0.53) and plant richness (0.57). Warming delayed FFR via a decrease in SM (0.24), but degradation directly advanced FFR (−0.17). MAP indirectly advanced OLO (−0.19) through a decrease in ST and directly advanced the onsets of other phenophases (Figure 6a,b). Strong positive covariances among onsets of all phenological stages (0.45–0.74) indicated tight phenological synchrony (Figure 6a).

**FIGURE 6:**
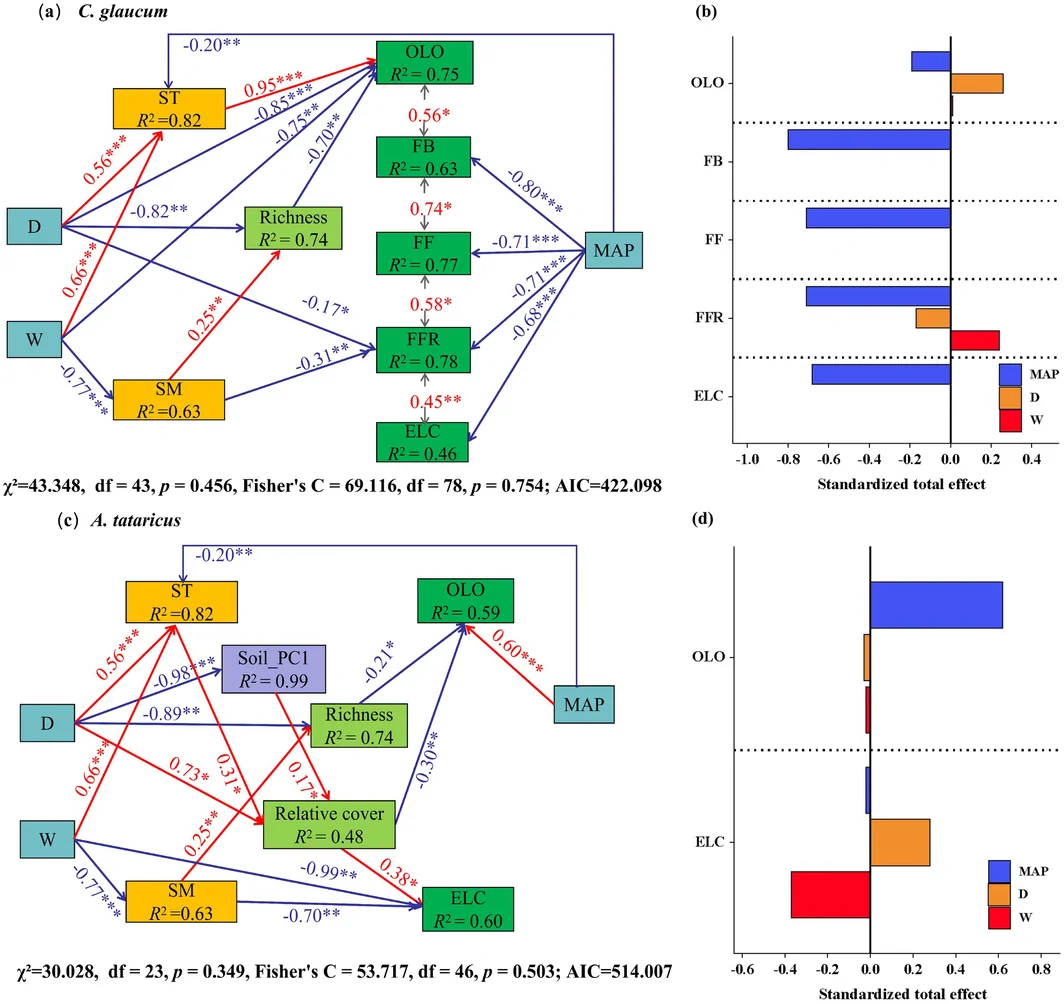
Structural equation models examining the direct and indirect effects of warming (W), degradation (D), and mean annual precipitation (MAP) on the onsets of phenological events for *C. glaucum* and *A. tataricus*. Values adjacent to arrows are standardized path coefficients (*p < 0.05; **p < 0.01; ***p < 0.001). Red and blue arrows indicate positive and negative effects, respectively, while gray double‐headed arrows denote covariances between variables. Proportions of variance explained (*R* ^2^) are shown below each endogenous variable. (a) Onset models for *C. glaucum*; (b) Standardized total effects on phenological onset for *C. glaucum*; (c) Onset models for *A. tataricus*; (d) Standardized total effects on phenological onset for *A. tataricus*.

For *A. tataricus* (Figure 6c,d), the total net effect of warming on OLO was small (−0.02) due to the offsetting effects of an indirect advance via SM and richness (0.04) and a delay via ST and relative cover (−0.06). However, it had a net advance effect on leaf coloring (−0.37) via a direct advance effect (−0.99) and an indirect delay effect via reduced SM (0.54) and increased ST (0.08). Degradation had a net small effect on OLO (−0.03) through the indirectly delay effect of plant richness and soil quality (0.24) and through the advance effect of relative coverage (−0.27). It had a net delay effect on leaf coloring (0.29) via an indirect delay of relative coverage (0.35) and an indirect advance of soil quality (−0.06). Moreover, precipitation had a net delay effect on OLO (0.62) via direct (0.60) and indirect effects of relative cover (0.02), whereas it had a small indirect effect on leaf coloring (−0.02) via decreased soil temperature and relative coverage. There was no significant covariance between OLO and leaf‐coloring (Figure 6c).

For *C. glaucum* phenological duration (Figure 7a,b), warming strongly shortened leafing duration (DL; total −1.44) through direct (−0.82) and indirect effects via ST (−0.62). Degradation overall shortened DL (−0.25) via a direct prolonging effect (0.87) and indirect shortening effects of ST (−0.53) and plant richness (−0.59). Precipitation indirectly prolonged DL (0.19) via ST. Budding duration (DB) was indirectly shortened by warming (−0.15) and degradation (−0.13) via increased ST. Flowering duration (DF) was indirectly shortened by warming (−0.24) and degradation (−0.20) via increased ST, and precipitation had a net prolonging effect on it (−0.38) through direct (−0.45) and indirect effects of decreased ST (0.07). Similarly, fruiting duration (DFR) was prolonged by warming (0.51) and degradation (0.43) via increased ST, and precipitation had a net prolonging effect on it (0.48) through direct (0.63) and indirect effects (−0.15). Leaf‐coloring duration (DLC) was prolonged directly by degradation (0.38). Total active period (TAP) was strongly shortened by degradation (−1.78) via direct (−0.83) and indirect effects of ST (−0.45) and plant richness (−0.50). Warming had a net shortening effect on TAP (−1.19) via direct (−0.36) and indirect effects of increased ST (−0.53) and decreased SM (−0.30). Precipitation had an indirect effect on it via decreased ST (0.16).

**FIGURE 7:**
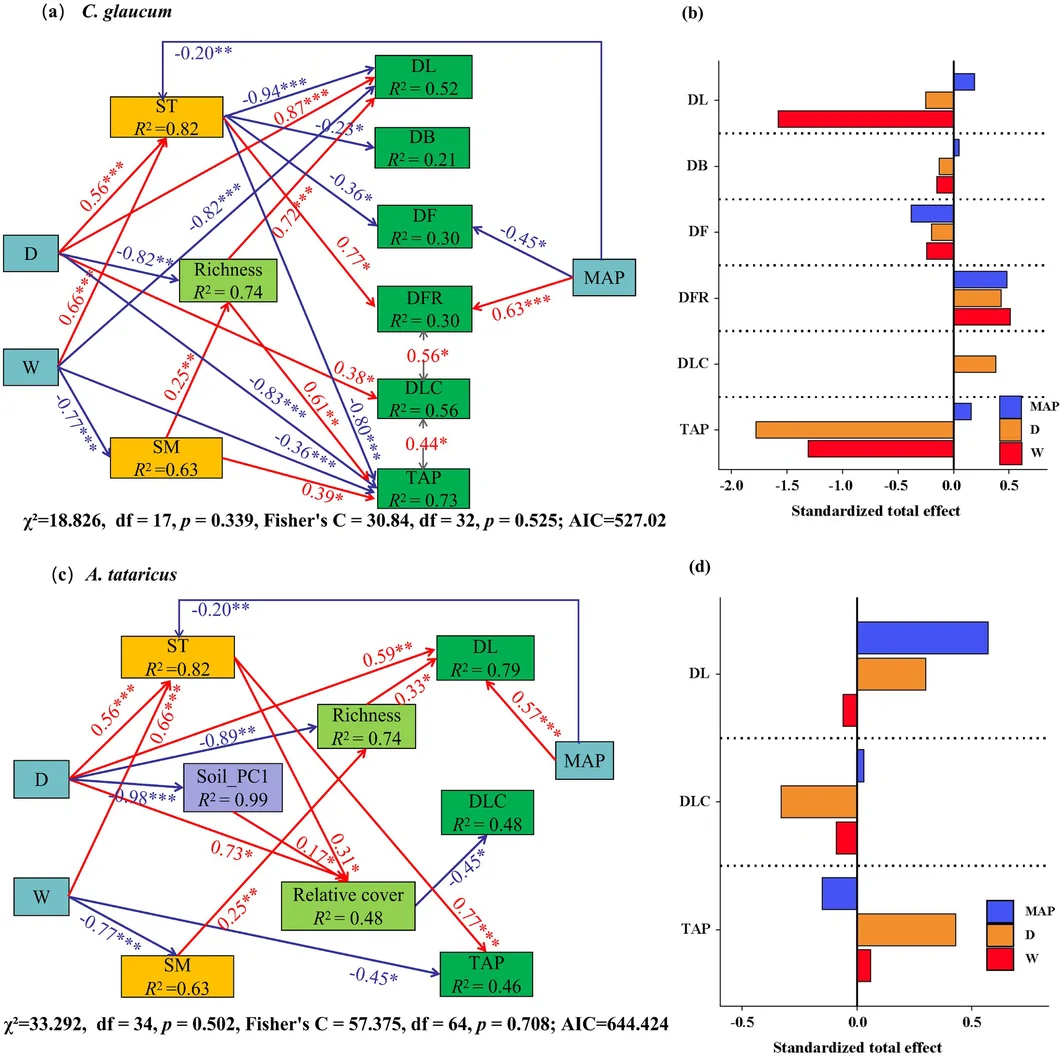
Structural equation models examining the direct and indirect effects of warming (W), degradation (D), and mean annual precipitation (MAP) on the duration of phenological events for *C. glaucum* and *A. tataricus*. Values adjacent to arrows are standardized path coefficients (*p < 0.05; **p < 0.01; ***p < 0.001). Red and blue arrows indicate positive and negative effects, respectively, while gray double‐headed arrows denote covariances between variables. Proportions of variance explained (*R* ^2^) are shown below each endogenous variable. (a) Duration models for *C. glaucum*; (b) Standardized total effects on phenological duration for *C. glaucum*; (c) Duration models for *A. tataricus*; (d) Standardized total effects on phenological duration for *A. tataricus*.

For *A. tataricus* phenological duration (Figure 7c,d), warming had a small net shortening effect on DL (−0.06) via decreased SM and plant richness. Degradation had a net prolonging effect on DL (0.30) through the direct (0.59) and indirect effect of reduced richness (−0.29). Precipitation directly prolonged DL (0.57). DLC was indirectly shortened by degradation via increased relative coverage (−0.33) and ST (−0.08) and decreased soil quality (0.07). Warming had an indirect shortening effect on it (−0.09) through increased ST and relative cover. TAP was indirectly prolonged by degradation (0.43) through increased ST, and warming had a net indirect effect on it (0.06) via a direct (−0.45) and indirect effect of increased ST (0.51).

## 4 Discussion

Overall, warming, degradation, and their interaction did not alter the mean onsets and durations of phenophases or leaf‐coloring for the annual plant (Figure 2a,c), although their effects depended on the year (Figure 3). By contrast, severe degradation advanced the onset of the perennial *A. tataricus* and prolonged its total active period relative to moderate degradation under warming, with strong interannual variation (Figures 2b,d, 3b,d). These results supported our hypotheses that the perennial plant would be more sensitive to warming relative to the annual plant from the perspective of plant phenology, and there was an interactive effect between warming and degradation on perennial plant phenology. Moreover, precipitation regulated the responses of the phenophases of annual and perennial plants to warming and degradation.

### 4.1 Effects of Warming and Degradation on Annual Plant Phenology and the Regulatory Role of Precipitation

Some studies have shown that precipitation was a main cue for germination and development in alpine annual plants (Cui et al. 2017; Suonan et al. 2021; Zhang et al. 2025). Multi‐year means could mask real treatment effects because positive and negative anomalies cancel across wet and dry years (Chen et al. 2019; Hong et al. 2023). Our results also indicated that annual mean precipitation had the strong direct effect on *C. glaucum* phenology (Figure 6b). For example, annual precipitation was normal in 2014 and dry in 2015, and warming reduced soil moisture by 20%–36% in the alpine grassland during April–May (Cui et al. 2017; Dai et al. 2020; Zhou et al. 2024). Warming delayed first fruiting of the annual plant under moderate degradation (Figure 3a; Table S3) in 2014 and 2015, because pre‐season drought and decreased soil moisture induced by warming co‐limited its reproduction during budding‐to‐fruiting phenology (Facelli and Chesson 2008; Mayfield et al. 2014; Liu et al. 2022). However, warming alone did not affect the mean onsets of any of the phenophases of the annual plant in 2016 (Figure 3a; Table 1) because it was a wet year (Zhou et al. 2024). This could be due to the high precipitation offsetting the negative effects of decreased soil moisture induced by warming. Moreover, warming alone did not affect the onsets of phenophase for severe degradation during the experimental period (Figure 3a; Table 1; Table S3), whereas MD significantly advanced first flowering relative to SD under warming, but not under no‐warming in 2016 (Figure 3a), suggesting that there was an interaction between warming and degradation. For example, degradation alone (e.g., NWSD vs. NWMD) did not affect onsets of phenophases (Table S3), whereas WSD significantly delayed first flowering compared with NWMD in 2016 (Figure 3a). This could be for three reasons. First, severe degradation reduced soil nitrogen and plant diversity (Figure S3; Figure 4b). Nitrogen limitation dampens phenological plasticity (Wang and Tang 2019; Bektaş et al. 2021; Liu et al. 2022; Zhang et al. 2026). Second, reduced species richness (Figure 4b) also weakens phenological sensitivity to temperature (Wolf et al. 2017; Peng et al. 2022). Thus, the negative effects of poor soil fertility and low diversity could override any positive warming effects (Cui et al. 2017; Bektaş et al. 2021; Zhou et al. 2024; Chen et al. 2025). Third, severe degradation reduced soil water‐holding capacity and organic matter, and even mild warming‐induced soil moisture reduction (15.4%–18.8% in SD) caused hidden water stress during flowering, consistent with degradation amplifying drought sensitivity (Zhu et al. 2016; Ma et al. 2023).

In addition, severe degradation (SD) prolonged leaf‐coloring duration (DLC) relative to moderate degradation (MD) under no‐warming in 2014, whereas it also prolonged DLC and total activity period (TAP) compared to MD under warming in 2016 (Figure 3c). Some studies have shown that warming accelerated leaf senescence (Fu et al. 2018; Guo et al. 2026) or prolonged leaf senescence or TAP (Li et al. 2016) depending on soil moisture. Moreover, nutrient depletion and water stress in severely degraded plots could suppress leaf senescence, thereby prolonging TAP due to longer photosynthesis to compensate for carbon loss (Wang et al. 2012; Jiang et al. 2016). Noting that 2014 and 2016 were normal or wet years (Zhou et al. 2024), high precipitation could mediate the drought effect of warming on growing season length (Liu et al. 2021; Ma et al. 2023) because phenological transitions could be primarily controlled by water availability (Suonan et al. 2021; Cui et al. 2017).

### 4.2 Effects of Warming and Degradation on Perennial Plant Phenology and the Regulatory Role of Precipitation

Although many studies show that warming advances perennial leaf‐out by 2–14 days (Wolkovich et al. 2012; Wang et al. 2014; Jiang et al. 2016), our study found no significant three‐year mean warming effect on *A. tataricus* leaf‐out. Instead, leaf‐out showed strong interannual variation. In the dry year 2015 (spring precipitation 79 mm), warming reduced soil moisture (Cui et al. 2017) but significantly advanced leaf‐out in severely degraded (SD) plots. This could be attributed to the low vegetation cover and rapid soil warming in SD plots. Although water deficit usually delays leaf‐out (Liu et al. 2025), the positive effect of warming outweighed the negative drought effect, resulting in earlier leaf‐out. However, in the wet year 2016 (spring precipitation 99 mm, 20.2% higher than 2015) (Cui et al. 2017), warming significantly delayed leaf‐out in moderately degraded (MD) plots. This delay can be explained by two mechanisms. First, the legacy effect of the previous dry year (2015) may have delayed leaf‐out in 2016, as water deficit in drylands can have strong carry‐over effects on phenology (Liu et al. 2025). Second, water conditions and warming may promote nitrogen mineralization, increasing nitrogen availability in MD plots. A meta‐analysis showed that nitrogen availability generally delays phenological timing (Wang and Tang 2019). Moreover, increased species richness in MD plots may intensify competition with other herbaceous plants (especially annuals), further delaying leaf‐out (Zhang et al. 2020). Thus, the net effect of warming on leaf‐out is a non‐linear response co‐regulated by spring hydrothermal conditions and degradation status. Degradation alone (NWSD vs. NWMD) did not significantly change leaf‐out or leaf‐coloring dates (Figure 3b; Table S3). However, when combined with warming, leaf‐out was significantly advanced in both 2014 and 2015 (WSD vs. NWMD). This indicates that degradation itself does not directly alter phenological onset, but modulates warming effects by changing canopy structure, soil microclimate, and nitrogen availability. Previous studies have often indicated a process in which warming intensified spring drought which in turn delayed phenology for annual plants (Cui et al. 2017; Hong et al. 2023). In contrast to such studies, our study observed earlier leaf‐out of a perennial under dry conditions, highlighting the roles of life‐history strategy and degradation state.

Phenological durations did not always respond synchronously with onset dates. Degradation alone significantly extended DL of the perennial plant in 2014 and 2015, but shortened DLC in 2014 (Table 2; Table S4). SD significantly extended total active period (TAP) under warming in 2014 because spring warming extends DL by advancing leaf‐out, while autumn warming extends DLC by delaying senescence (Luo et al. 2010; Hong et al. 2023). The effect of warming on leaf senescence is phase‐dependent: warming accelerates senescence before the summer solstice, whereas it slows senescence after the solstice (Zohner et al. 2023; Qiu et al. 2024). High temperature or soil drought during mid‐to‐late growing season shortens DLC and accelerates senescence (Hong et al. 2023). In the dry year 2015, no significant treatment effect on DLC was observed, likely because drought already dominated senescence, masking warming effects (Liu et al. 2025). Warming can first delay and then accelerate leaf senescence, leading to bidirectional DLC changes (Shi et al. 2014). Thus, warming and degradation jointly control DL and DLC, but their effects strongly depend on interannual variation in water and temperature.

Precipitation mediates the direction of warming and degradation effects. Under dry conditions (2015), severe degradation amplified warming‐induced leaf‐out advancement, while under wet conditions (2016), warming delayed leaf‐out in moderately degraded plots. Thus, water limitation strengthens the amplifying effect of degradation on warming, while ample water weakens or reverses it. Therefore, precipitation is a key regulator, and the direction of its effect depends on life‐history strategy (Ma et al. 2023, 2025). These results further support the finding that degraded grasslands amplify perennial plants' responses to warming in terms of earlier leaf‐out and longer total active period, though the effect on leaf coloring may be inconsistent (Li et al. 2019; Zhu et al. 2016).

### 4.3 Distinct Life‐History Strategies Determine the Contrasting Phenological Responses of Annual and Perennial Forbs to Warming and Degradation

The contrasting phenological responses of annual and perennial forbs arise from fundamental differences in life‐history strategies and resource allocation. Annuals follow a bet‐hedging strategy, prioritizing rapid reproduction within a single growing season (Childs et al. 2010; Satake 2010; Vico et al. 2016). This favors a conservative phenological response to environmental fluctuations, as reflected in our finding that warming and degradation had limited mean effects on annual phenophases. Perennials adopt a repeated reproductive strategy, spreading reproductive effort across multiple years, which allows for greater phenological plasticity (Hyun et al. 2019). This explains why perennials exhibited higher sensitivity to warming and degradation, with advanced leaf‐out and extended total active period under severe degradation combined with warming.

Resource allocation further differentiates the two groups. Perennials possess well‐developed root systems and carbohydrate reserves that buffer them against environmental stress, enabling greater phenological adjustment when conditions improve (Shi et al. 2015). Annuals lack such storage organs and rely entirely on current‐season resource acquisition, making them vulnerable to degradation‐induced resource limitation (Cui et al. 2017; Suonan et al. 2021). This dependency overrides the positive effects of warming and results in phenological stasis or delay when soil nitrogen and water availability are reduced.

Precipitation modulates these differential responses through distinct mechanisms (Liu et al. 2017). For annuals, precipitation primarily affects germination and seedling establishment, explaining the strong direct effect of annual mean precipitation on *C. glaucum* phenology and the delay of reproductive events under drought. For perennials, precipitation interacts with warming and degradation through soil moisture and nutrient availability. Our finding that precipitation mediated the direction of warming effects—amplifying advancement under dry conditions (2015) but reversing it under wet conditions (2016)—demonstrates that perennials integrate moisture signals with thermal cues to determine optimal phenological timing. These fundamental differences have important implications for predicting plant community dynamics under future climate change.

### 4.4 Limitations and Future Directions

Several limitations of this study should be acknowledged. First, our experiment was conducted at a single site in Naqu on the central Qinghai‐Xizang Plateau, which limits the spatial generality of our findings (Sun et al. 2025). Future studies should extend this experimental framework to multiple sites across the Plateau to assess the spatial heterogeneity of phenological responses.

Second, our study focused on only two forb species—the annual *C. glaucum* and the perennial *A. tataricus* . Different functional groups exhibit divergent phenological responses to environmental changes, and community‐level dynamics cannot be fully captured by examining only two species (Liu et al. 2017). We therefore caution against extrapolating our results to ecosystem‐level restoration or community reassembly.

Third, soil physicochemical properties were measured only in 2016, which precluded separate SEM analyses for each year. While our LMMs confirmed significant year × treatment interactions, annual SEMs would provide more direct mechanistic understanding. Future studies should include multi‐year soil monitoring to enable such analyses.

Fourth, our experiment was conducted over three growing seasons, which may not capture longer‐term demographic responses. Phenological shifts can have cumulative effects on population dynamics that only become apparent over longer time scales (Ma et al. 2020). Long‐term monitoring is needed to assess the persistence of the phenological changes we observed.

To address these limitations, we propose: (1) multi‐site experiments across the Qinghai‐Xizang Plateau to test the generality of our findings; (2) inclusion of broader plant functional types and community‐level metrics; (3) integration of high‐frequency environmental monitoring for year‐specific mechanistic modeling; and (4) long‐term demographic and seed bank studies to evaluate population‐level impacts.

## 5 Conclusions

Our results showed that warming and degradation generally did not affect the phenophases of the annual *C. glaucum* , although their effects varied with year. Desertified grassland advanced the onset of leaf‐out of the perennial *A. tataricus* and prolonged its total active period relative to moderately degraded grassland under warming. Moreover, precipitation primarily determined the phenophases of the annual plant, and it regulated the phenological responses of the annual and perennial plants to warming and degradation through altered soil microclimate and plant community structure. These findings suggest that warming may promote the growth and reproduction of perennial forbs (specifically *A. tataricus* ) in alpine desertified grasslands, potentially contributing to the recovery of forb components in degraded alpine meadows. However, we emphasize that this inference is limited to the forb functional group and our single study site; whether such responses translate to ecosystem‐level restoration—which would require concurrent recovery of grasses, sedges, and overall community structure—remains an open question that warrants further investigation.

## Author Contributions

**Lanying Chen:** conceptualization (equal), data curation (equal), methodology (equal), writing – original draft (equal), writing – review and editing (equal). **Shujuan Cui:** data curation (equal), investigation (equal), methodology (equal). **Jianping Sun:** writing – review and editing (supporting). **Wangwang Lv:** writing – review and editing (supporting). **Jingya Lv:** writing – review and editing (supporting). **Fang Yuan:** writing – review and editing (supporting). **Yang Zhou:** writing – review and editing (supporting). **A. Wang:** writing – review and editing (supporting). **Shiping Wang:** conceptualization (lead), writing – review and editing (equal).

## Funding

This work was supported by the National Natural Science Foundation of China (42230504), the Lhasa Science and Technology Plan Project (LSKJ202622), the Science and Technology Project of Xizang Autonomous Region (XZ202601JD0005), the Young Scientists Fund of the National Natural Science Foundation of China (42507348), the China Postdoctoral Science Foundation (GZB20240779), and the High‐level Graduate Talent Cultivation Program of Xizang University (2025‐GSP‐B013).

## Conflicts of Interest

The authors declare no conflicts of interest.

## Supporting information


**Table S1:** Results of two‐way ANOVA testing the effects of degradation (D), warming (W), and their interaction (D × W) on soil physicochemical properties measured in 2016.
**Table S2:** Results of linear mixed‐effects models (LMMs) testing the effects of degradation (D), warming (W), and their interaction (W × D) on species richness and relative cover of *C. glaucum* and *A. tataricus* .
**Table S3:** Mean (±SE) of phenological dates (Julian day of year, DOY) for *A. tataricus* and *C. glaucum* under different treatments (NWMD, WMD, NWSD, WSD) from 2014 to 2016 and the three–year mean.
**Table S4:** Mean (±SE) of phenological durations for *A. tataricus* and *C. glaucum* under different treatments (NWMD, WMD, NWSD, WSD) from 2014 to 2016 and the three–year mean.
**Figure S1:** Dynamics of daily mean air temperature (AT) (°C) and daily total precipitation (mm) from 2014 to 2016.
**Figure S2:** Correlation matrix among soil and climatic variables (a) and PCA biplots for soil physicochemical properties (b).
**Figure S3:** Average soil physicochemical properties in NWMD, WMD, NWSD, and WSD treatments in 2016.

## Data Availability

Data are provided as private‐for‐peer review via the following link: https://figshare.com/s/0aab4c743cf841762131.
