# Supplementary material for: Precipitation Regulates Phenological Responses of Annual and Perennial Forbs to Warming and Degradation in an Alpine Meadow on the Qinghai–Xizang Plateau
Source: Ecol Evol. 2026 Sep 29;16(10):e74423. doi: 10.1002/ece3.74423 (PMC13621482; doi:10.1002/ece3.74423)
Supplement: Supplementary file 1 [file ECE3-16-e74423-s001.docx]

**Supplementary materials**

**FIGURE S1** Dynamics of daily mean air temperature (AT) (℃) and daily total precipitation (mm) from 2014 to 2016.

**FIGURE S2** Correlation matrix among soil and climatic variables (a) and PCA biplots for soil physicochemical properties (b). Abbreviations: TN = Total nitrogen, TP = Total phosphorus, AP = Available phosphorus, AN = Available nitrogen, TOC = Total organic carbon, TC = Total carbon, AK = Available potassium, TK = Total potassium, AirT=Air temperature, AirP=Air precipitation. Absolute PC1 Loading reflects the contribution of each indicator to PC1; higher values indicate greater contribution.

**FIGURE S3** Average soil physicochemical properties in NWMD, WMD, NWSD, and WSD treatments in 2016. Different letters indicate significant difference at the 0.05 level. Mean±SE is shown in the figure.

**TABLE S1 Results of two‑way ANOVA testing the effects of degradation (D), warming (W), and their interaction (D×W) on soil physicochemical properties measured in 2016.**

| **Soil variable** | **Source** | ***F*-value** | ***p*-value** |
| --- | --- | --- | --- |
| pH value | Degradation (D) | 25.22 | **<0.001** |
|  | Warming (W) | 2.33 | 0.153 |
|  | D×W | 0.63 | 0.444 |
| TOC (%) | D | 166.55 | **<0.001** |
|  | W | 0.19 | 0.674 |
|  | D×W | 0.26 | 0.622 |
| TN (g·kg⁻¹) | D | 322.66 | **<0.001** |
|  | W | 0.16 | 0.694 |
|  | D×W | 0.03 | 0.869 |
| TP (g·kg⁻¹) | D | 745.60 | **<0.001** |
|  | W | 0.02 | 0.898 |
|  | D×W | 0.24 | 0.633 |
| TK (g·kg⁻¹) | D | 88.43 | **<0.001** |
|  | W | 0.002 | 0.968 |
|  | D×W | 0.32 | 0.579 |
| AN (mg·kg⁻¹) | D | 564.21 | **<0.001** |
|  | W | 0.05 | 0.829 |
|  | D×W | 0.05 | 0.834 |
| AP (mg·kg⁻¹) | D | 1061.31 | **<0.001** |
|  | W | <0.001 | 0.990 |
|  | D×W | 0.11 | 0.745 |
| AK (mg·kg⁻¹) | D | 164.00 | **<0.001** |
|  | W | 0.20 | 0.666 |
|  | D×W | 0.02 | 0.904 |

Note: D = degradation (moderately degraded, MD; severely degraded, SD); W = warming (no‑warming, NW; warming, W). Significant *p*‑values (< 0.05) are shown in bold. Two‑way ANOVA was conducted with degradation and warming as fixed factors.

**TABLE S2** Results of linear mixed-effects models (LMMs) testing the effects of degradation (D), warming (W), and their interaction (W×D) on species richness and relative cover of *A. tataricus* and *C. glaucum*.

| **Effect** | **Species richness** | | A. **tataricus**   **relative cover** | | B. **glaucum**   **relative cover** | |
| --- | --- | --- | --- | --- | --- | --- |
|  | **F-value** | **p-value** | **F-value** | **p-value** | **F-value** | **p-value** |
| Year (Y) | 5.29 | **0.010** | 0.76 | 0.471 | 1.17 | 0.318 |
| Warming (W) | 4.32 | **0.045** | 0.64 | 0.429 | 7.16 | **0.009** |
| Degradation (D) | 85.75 | **<0.001** | 13.22 | **<0.001** | 3.87 | 0.053 |
| Y× W | 0.57 | 0.570 | 0.07 | 0.934 | 0.14 | 0.874 |
| Y × D | 0.25 | 0.780 | 0.77 | 0.466 | 0.65 | 0.524 |
| W × D | 6.04 | **0.019** | 0.13 | 0.725 | 0.67 | 0.417 |
| Y × W × D | 0.68 | 0.514 | 0.28 | 0.755 | 0.12 | 0.886 |

Note: D = degradation (moderately degraded, MD; severely degraded, SD); W = warming (no‑warming, NW; warming, W). Significant *p*‑values (< 0.05) are shown in bold. Plot was included as a random intercept to account for repeated measurements across years; year was treated as a random factor.

**TABLE S3** Mean (±SE) of phenological dates (Julian day of year, DOY) for *A. tataricus* and *C. glaucum* under different treatments (NWMD, WMD, NWSD, WSD) from 2014 to 2016 and the three-year mean.

| Species | Year | Phenology | NWMD | WMD | NWSD | WSD |
| --- | --- | --- | --- | --- | --- | --- |
| *A. tataricus* | 2014 | OLO | 161.5 ± 1.9ab | 164.2 ± 3.4a | 154.0 ± 0.0bc | 151.8 ± 2.2c |
| *A. tataricus* | 2014 | ELC | 223.0 ± 3.9a | 229.5 ± 4.5a | 232.5 ± 4.2a | 227.5 ± 3.8a |
| *A. tataricus* | 2015 | OLO | 151.2 ± 2.7a | 149.0 ± 1.9ab | 153.5 ± 2.9a | 141.0 ± 1.0b |
| *A. tataricus* | 2015 | ELC | 242.8 ± 10.7a | 243.2 ± 8.8a | 270.0 ± 3.5a | 236.0 ± 7.9a |
| *A. tataricus* | 2016 | OLO | 164.5 ± 1.4b | 180.5 ± 1.4a | 164.0 ± 1.7b | 164.0 ± 1.7b |
| *A. tataricus* | 2016 | ELC | 247.2 ± 4.0a | 248.2 ± 3.7a | 242.0 ± 0.0a | 238.5 ± 4.9a |
| *A. tataricus* | Mean | OLO | 159.1 ± 2.0ab | 164.6 ± 4.1a | 157.2 ± 1.8ab | 152.2 ± 3.0b |
| *A. tataricus* | Mean | ELC | 237.7 ± 4.8a | 240.3 ± 4.0a | 248.2 ± 5.1a | 234.0 ± 3.4a |
| *C. glaucum* | 2014 | OLO | 159.2 ± 2.6a | 160.0 ± 2.6a | 156.2 ± 3.1a | 160.0 ± 2.1a |
| *C. glaucum* | 2014 | FB | 202.0 ± 4.7a | 209.8 ± 2.2a | 211.5 ± 4.4a | 208.0 ± 5.4a |
| *C. glaucum* | 2014 | FF | 214.5 ± 4.8a | 228.2 ± 3.5a | 230.5 ± 4.3a | 221.5 ± 6.2a |
| *C. glaucum* | 2014 | FFR | 223.5 ± 4.1b | 238.2 ± 3.4a | 225.8 ± 1.7ab | 231.5 ± 2.6ab |
| *C. glaucum* | 2014 | ELC | 237.5 ± 2.0a | 241.0 ± 0.0a | 241.0 ± 0.0a | 237.5 ± 2.0a |
| *C. glaucum* | 2015 | OLO | 179.5 ± 4.5a | 177.0 ± 3.5a | 169.0 ± 2.0a | 176.8 ± 4.0a |
| *C. glaucum* | 2015 | FB | 221.0 ± 0.0a | 226.8 ± 4.0a | 224.0 ± 1.6a | 224.5 ± 1.4a |
| *C. glaucum* | 2015 | FF | 234.5 ± 3.4a | 245.2 ± 2.6a | 243.5 ± 3.2a | 238.2 ± 2.2a |
| *C. glaucum* | 2015 | FFR | 243.8 ± 3.8b | 255.0 ± 2.1a | 253.2 ± 1.8ab | 249.0 ± 0.0ab |
| *C. glaucum* | 2015 | ELC | 259.5 ± 1.5a | 260.2 ± 9.3a | 244.8 ± 5.1a | 259.0 ± 5.0a |
| *C. glaucum* | 2016 | OLO | 175.2 ± 2.8a | 174.5 ± 2.0a | 182.2 ± 2.8a | 175.8 ± 2.9a |
| *C. glaucum* | 2016 | FB | 202.2 ± 1.2ab | 197.0 ± 2.6b | 204.8 ± 1.2a | 207.5 ± 1.5a |
| *C. glaucum* | 2016 | FF | 209.0 ± 1.7bc | 202.2 ± 2.4c | 219.2 ± 1.2ab | 227.0 ± 5.7a |
| *C. glaucum* | 2016 | FFR | 221.0 ± 5.2a | 223.5 ± 8.4a | 231.5 ± 1.5a | 237.0 ± 4.1a |
| *C. glaucum* | 2016 | ELC | 239.0 ± 1.7a | 239.0 ± 1.7a | 237.5 ± 1.5a | 239.0 ± 1.7a |
| *C. glaucum* | Mean | OLO | 171.3 ± 3.2a | 170.5 ± 2.7a | 169.2 ± 3.5a | 170.8 ± 2.8a |
| *C. glaucum* | Mean | FB | 208.4 ± 3.1a | 211.2 ± 4.0a | 213.4 ± 2.8a | 213.3 ± 3.0a |
| *C. glaucum* | Mean | FF | 219.3 ± 3.8a | 225.2 ± 5.5a | 231.1 ± 3.4a | 228.9 ± 3.4a |
| *C. glaucum* | Mean | FFR | 229.4 ± 3.8a | 238.9 ± 4.8a | 236.8 ± 3.7a | 239.2 ± 2.6a |
| *C. glaucum* | Mean | ELC | 245.3 ± 3.2a | 246.8 ± 4.1a | 241.1 ± 1.8a | 245.2 ± 3.4a |

Note: Different lowercase letters within the same row indicate significant differences among treatments at the 0.05 level (Tukey’s HSD test).

**TABLE S4** Mean (±SE) of phenological durations for *A. tataricus* and *C. glaucum* under different treatments (NWMD, WMD, NWSD, WSD) from 2014 to 2016 and the three‑year mean.

| Species | Year | Phenology | NWMD | WMD | NWSD | WSD |
| --- | --- | --- | --- | --- | --- | --- |
| *A. tataricus* | 2014 | DL | 47.0 ± 4.7b | 42.8 ± 1.4b | 62.0 ± 0.0a | 72.5 ± 2.4a |
| *A. tataricus* | 2014 | DLC | 45.0 ± 2.0a | 38.0 ± 1.8a | 22.0 ± 1.2b | 20.0 ± 2.0b |
| *A. tataricus* | 2014 | TAP | 128.2 ± 0.9b | 120.5 ± 2.1c | 130.8 ± 1.2b | 136.8 ± 0.9a |
| *A. tataricus* | 2015 | DL | 9.0 ± 0.4b | 12.8 ± 1.7ab | 16.8 ± 2.8a | 11.5 ± 0.9ab |
| *A. tataricus* | 2015 | DLC | 13.8 ± 1.8b | 16.2 ± 3.2ab | 18.2 ± 2.0ab | 25.8 ± 2.7a |
| *A. tataricus* | 2015 | TAP | 132.8 ± 3.4a | 136.0 ± 4.1a | 139.5 ± 3.6a | 145.2 ± 3.0a |
| *A. tataricus* | 2016 | DL | 23.8 ± 4.6a | 24.8 ± 3.6a | 24.0 ± 4.0a | 27.5 ± 1.5a |
| *A. tataricus* | 2016 | DLC | 26.8 ± 5.7a | 30.8 ± 6.7a | 26.0 ± 6.6a | 42.2 ± 3.1a |
| *A. tataricus* | 2016 | TAP | 135.5 ± 3.1a | 123.2 ± 8.3a | 127.5 ± 2.9a | 139.5 ± 2.9a |
| *A. tataricus* | Mean | DL | 26.6 ± 5.1a | 26.8 ± 3.9a | 34.2 ± 6.2a | 37.2 ± 7.8a |
| *A. tataricus* | Mean | DLC | 28.5 ± 4.3a | 28.3 ± 3.6a | 22.1 ± 2.3a | 29.3 ± 3.2a |
| *A. tataricus* | Mean | TAP | 132.2 ± 1.7ab | 126.6 ± 3.5b | 132.6 ± 2.1ab | 140.5 ± 1.7a |
| *C. glaucum* | 2014 | DL | 50.8 ± 3.9a | 44.0 ± 4.1a | 57.5 ± 0.6a | 54.2 ± 4.6a |
| *C. glaucum* | 2014 | DB | 17.5 ± 3.4ab | 21.2 ± 3.2a | 18.8 ± 0.8a | 7.8 ± 1.7b |
| *C. glaucum* | 2014 | DF | 13.2 ± 1.9a | 12.8 ± 2.6a | 10.0 ± 0.6a | 6.5 ± 0.6a |
| *C. glaucum* | 2014 | DFR | 18.2 ± 2.4a | 9.5 ± 2.4a | 16.2 ± 2.6a | 16.5 ± 4.6a |
| *C. glaucum* | 2014 | DLC | 26.2 ± 3.8b | 27.5 ± 1.4ab | 38.8 ± 2.4a | 37.5 ± 3.2ab |
| *C. glaucum* | 2014 | TAP | 136.8 ± 2.6a | 136.0 ± 2.6a | 137.2 ± 3.6a | 132.2 ± 0.5a |
| *C. glaucum* | 2015 | DL | 10.0 ± 3.2a | 16.5 ± 1.3a | 18.0 ± 2.5a | 11.8 ± 2.6a |
| *C. glaucum* | 2015 | DB | 19.8 ± 2.8a | 22.2 ± 4.0a | 19.8 ± 0.6a | 8.5 ± 1.4b |
| *C. glaucum* | 2015 | DF | 13.2 ± 2.8a | 12.8 ± 2.6a | 10.0 ± 0.6a | 6.8 ± 1.4a |
| *C. glaucum* | 2015 | DFR | 17.2 ± 2.6a | 9.0 ± 2.1b | 9.2 ± 1.2b | 9.0 ± 0.0b |
| *C. glaucum* | 2015 | DLC | 22.0 ± 4.9a | 28.2 ± 4.5a | 27.2 ± 3.3a | 18.2 ± 5.5a |
| *C. glaucum* | 2015 | TAP | 101.0 ± 7.2a | 117.0 ± 5.3a | 107.0 ± 2.0a | 100.5 ± 4.5a |
| *C. glaucum* | 2016 | DL | 14.5 ± 2.8a | 9.2 ± 1.6a | 14.8 ± 2.8a | 10.8 ± 1.2a |
| *C. glaucum* | 2016 | DB | 9.8 ± 1.6a | 12.0 ± 3.6a | 14.8 ± 3.1a | 22.5 ± 3.8a |
| *C. glaucum* | 2016 | DF | 12.0 ± 2.4a | 12.8 ± 3.4a | 16.8 ± 2.8a | 13.8 ± 4.4a |
| *C. glaucum* | 2016 | DFR | 15.5 ± 4.9a | 18.8 ± 3.8a | 15.5 ± 0.5a | 18.8 ± 4.5a |
| *C. glaucum* | 2016 | DLC | 12.5 ± 1.5b | 14.0 ± 6.7b | 18.2 ± 1.2b | 42.2 ± 2.9a |
| *C. glaucum* | 2016 | TAP | 105.2 ± 1.2b | 103.0 ± 3.1b | 93.8 ± 2.8b | 130.0 ± 4.4a |
| *C. glaucum* | Mean | DL | 25.1 ± 5.8a | 23.2 ± 4.7a | 30.1 ± 6.0a | 25.6 ± 6.3a |
| *C. glaucum* | Mean | DB | 15.7 ± 1.9a | 18.5 ± 2.3a | 17.8 ± 1.2a | 12.9 ± 2.4a |
| *C. glaucum* | Mean | DF | 12.8 ± 1.3a | 12.8 ± 1.5a | 12.2 ± 1.3a | 9.0 ± 1.7a |
| *C. glaucum* | Mean | DFR | 17.0 ± 1.8a | 12.4 ± 2.0a | 13.7 ± 1.3a | 14.8 ± 2.3a |
| *C. glaucum* | Mean | DLC | 20.2 ± 2.6b | 23.2 ± 3.2ab | 28.1 ± 2.8ab | 32.7 ± 3.8a |
| *C. glaucum* | Mean | TAP | 114.3 ± 5.3a | 118.7 ± 4.6a | 112.7 ± 5.7a | 120.9 ± 4.8a |

Note: Different lowercase letters within the same row indicate significant differences among treatments at the 0.05 level (Tukey’s HSD test).
